# Retinal ganglion cell loss in an ex vivo mouse model of optic nerve cut is prevented by curcumin treatment

**DOI:** 10.1038/s41420-021-00760-1

**Published:** 2021-12-15

**Authors:** Lucia Buccarello, Jessica Dragotto, Kambiz Hassanzadeh, Rita Maccarone, Massimo Corbo, Marco Feligioni

**Affiliations:** 1 EBRI Rita Levi-Montalcini Foundation, Rome, Italy; 2 Need Institute, Milan, Italy; 3 Department of Biotechnology and Applied Clinical Sciences, Health and Environmental Sciences, University of L’Aquila, L’Aquila, Italy; 4 Department of Neurorehabilitation Sciences, Casa Di Cura del Policlinico, Milan, Italy

**Keywords:** Cell death in the nervous system, Visual system

## Abstract

Retinal ganglion cell (RGC) loss is a pathologic feature common to several retinopathies associated to optic nerve damage, leading to visual loss and blindness. Although several scientific efforts have been spent to understand the molecular and cellular changes occurring in retinal degeneration, an effective therapy to counteract the retinal damage is still not available. Here we show that eyeballs, enucleated with the concomitant optic nerve cut (ONC), when kept in PBS for 24 h showed retinal and optic nerve degeneration. Examining retinas and optic nerves at different time points in a temporal window of 24 h, we found a thinning of some retinal layers especially RGC’s layer, observing a powerful RGC loss after 24 h correlated with an apoptotic, MAPKs and degradative pathways dysfunctions. Specifically, we detected a time-dependent increase of Caspase-3, -9 and pro-apoptotic marker levels, associated with a strong reduction of BRN3A and NeuN levels. Importantly, a powerful activation of JNK, c-Jun, and ERK signaling (MAPKs) were observed, correlated with a significant augmented SUMO-1 and UBC9 protein levels. The degradation signaling pathways was also altered, causing a significant decrease of ubiquitination level and an increased LC3B activation. Notably, it was also detected an augmented Tau protein level. Curcumin, a powerful antioxidant natural compound, prevented the alterations of apoptotic cascade, MAPKs, and SUMO-1 pathways and the degradation system, preserving the RGC survival and the retinal layer thickness. This ex vivo retinal degeneration model could be a useful method to study, in a short time window, the effect of neuroprotective tools like curcumin that could represent a potential treatment to contrast retinal cell death.

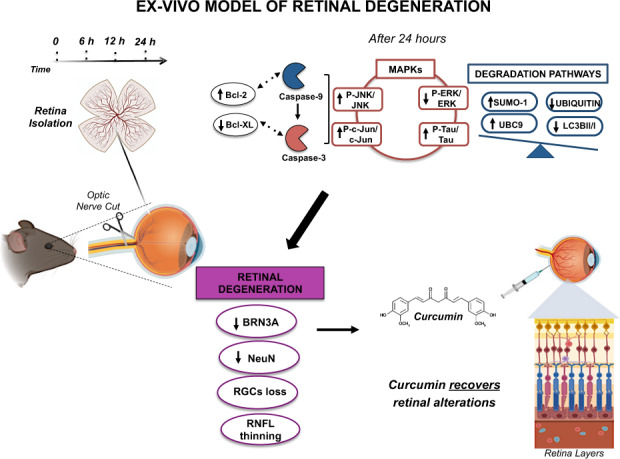

## Introduction

Retinal ganglion cell (RGC) loss [1] causes visual loss and blindness in several ocular pathologies. Retina is a neuronal tissue in which RGCs transfer the optical signals to the visual cortex in the brain which composes the images [2, 3]. An optic nerve damage induces progressive RGC apoptosis leading to blindness [4]. Pre-clinical studies in the retinal degeneration area are mostly pursued in using animal models, like glaucoma [5–7], in which RGC death is reproduced by several approaches, including the transient increase of intraocular pressure (IOP), optic nerve cut (ONC), or genetic manipulation [8–10]. The animal models are useful tools to investigate neuroprotective pharmacological treatments for visual system preservation [11] as well as in in vitro experiments, mostly based on RGC primary cultures, to test potential therapeutic compounds [12–14]. However, both in vivo and in vitro models do not fully recapitulate the human disease and showing invasiveness and reproducibility [15].

In this article, we performed an ex vivo model of ONC to test the neuroprotective effect of curcumin. ONC is obtained by enucleating the eyeballs and keeping the eyes in phosphate-buffered saline (PBS) for several hours at 4 °C. This causes a fast (24 h) RGC layer degeneration and other ganglion cell layer (GCL), inner plexiform layer (IPL), and inner nuclear layer (INL) thinning. So, this model might be useful for a preliminary screening of neuroprotective compounds with the advantage to reduce live animal pain and to be more physiological than in vitro approaches. Several molecular markers have been examined to study the degenerative processes such as the apoptotic pathway, already recognized as retinal degeneration marker [16–19], although the involvement of kinases and caspases has been quite controversial [20], and the pro/anti-apoptotic markers including caspases and BCL2 family, MAP-kinase pathways like JNK and ERK.

Recently, we reported that SUMO-1 plays an important role in the hyperactivation of JNK and Tau in a cellular model of oxidative stress and that curcumin, a strong antioxidant and anti-inflammatory natural molecule, was able to re-equilibrate the JNK–SUMO-1–Tau axis [21]. Since the involvement of SUMO in retinal function has been already shown [22], SUMO-1ylation levels in our ex vivo model has been investigated. In addition, photoreceptors and RGCs are known to be highly vulnerable to oxidative stress as that reactive oxidative species (ROS) imbalance is involved in many retinal diseases [23, 24]. Interestingly, curcumin ameliorates the retinal progressive damage by regulating the antioxidant system [25] and could represent an alternative treatment in retinal diseases [26]. Thus, the protective effect of curcumin has been tested in our ex vivo model.

We found that after 24 h from ONC and eyes enucleation induces a strong RGC death, optic nerve damage, and decrease of retina layers thickness and that curcumin pre-treatment is able to counteract the occurring retinal degeneration.

## Results

### The apoptotic pathway is activated in optic nerve and retina of enucleated mice eyeballs

**Fig. 1:**
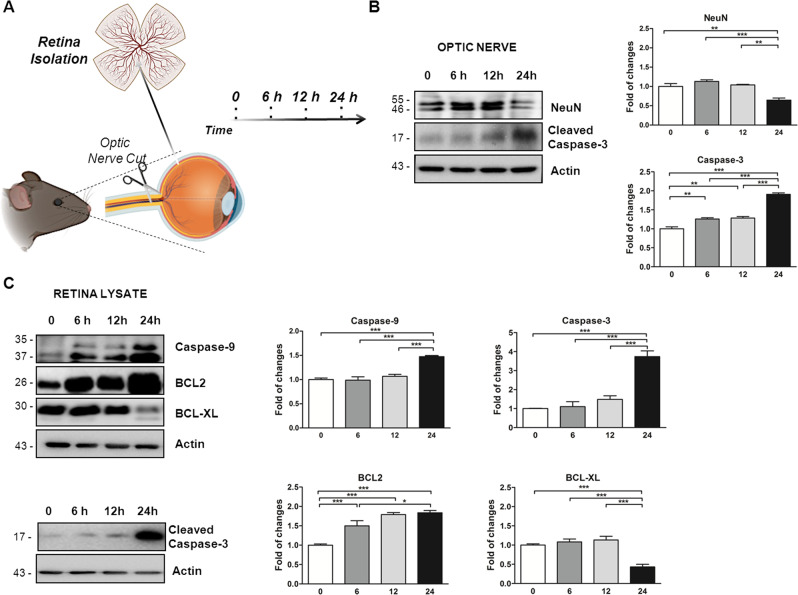
Activation of apoptotic pathway in the optic nerve and retina of C57BL/6J mice after 24 h from eye removal. **A** Graphical representation of experimental scheme performed to study the time-dependent retina neurodegeneration. After the eyeballs enucleation, optic nerves and retinal dissections were collected immediately after the sacrifice (time 0), after 6 (time 6), 12 (time 12), and 24 (time 24) hours from the sacrifice. **B** Representative western blots and relative quantifications of optic nerve lysates collected immediately after eye removal (time 0), after 6 h (time 6), 12 h (time 12), and 24 h (time 24). Graphs showed the significant time-dependent increase of cleaved-Caspase-3 level and decreased NeuN level in optic nerve lysates collected at time 24 in comparison to time 0. **C** Representative western blot and relative quantification of retinas lysates showed a significant augmented activation of Caspase-9, cleaved-Caspase-3, and BCL2 level at time 24 in comparison to time 0. The BCL-XL level was decreased in retinas collected at time 24 in comparison to time 0. Data were expressed as mean ± SEM. One-way ANOVA, Tukey’s post hoc test. *P < 0.05, **P < 0.01, and ***P < 0.001 [*n* = 5].

Enucleated mouse eyeballs, including ONC, kept in PBS for 24 h develops a progressive retinal degeneration resulting from the parameters analyzed in the retinas collected at different time points (time 0: *t*0; 6 h: *t*6; 12 h: *t*12, and 24 h: *t*24; Fig. 1A). Retinas and optic nerves lysates were examined by studying the apoptotic proteins, caspases, which are involved in RGC loss [27]. The cleaved-Caspase-3 level, active caspase effectors, was found increased twofolds, after 24 h from eye removal, in the optic nerve lysate (1.91 ± 0.06, *P* < 0.001 vs *t*0; Fig. 1B) and a lower but significant increase at time 6 (1.26 ± 0.06, *P* < 0.01 vs *t*0; Fig. 1B) and 12 (1.28 ± 0.06, *P* < 0.01 vs *t*0; Fig. 1B). Coherently, the expression level of a neuronal marker, NeuN, was strongly decreased at 24 h only (0.65 ± 0.07, *P* < 0.01 vs *t*0; Fig. 1B). In parallel, in the retina lysates Caspase-9 level, caspases initiator, was significantly increased in the retina lysates after 24 h (1.47 ± 0.05, *P* < 0.001 vs *t*0; Fig. 1C). In line, a significant increase of cleaved-Caspase-3 level was observed at time 24 (3.74 ± 0.23, *P* < 0.001 vs *t*0; Fig. 1C).

Other two major anti-apoptotic proteins of the BCL2 family, BCL2 and BCL-XL, were also measured. They play important roles in inhibiting mitochondria-dependent extrinsic and intrinsic cell death pathways [28]. The BCL2 level was significantly augmented during the 24 h starting after time 6 (1.50 ± 0.09, *P* < 0.001 vs *t*0; Fig. 1C) and 12 (1.79 ± 0.09, *P* < 0.001 vs *t*0; Fig. 1C) and reaching the maximal effect at time 24 (1.84 ± 0.08, *P* < 0.001 vs *t*0; Fig. 1C). In contrast, the BCL-XL level was decreased at time 24 (0.43 ± 0.08, *P* < 0.001 vs *t*0; Fig. 1C), underlying an important alteration of the anti-apoptotic pathway in retinas extracted after 24 h after eye removal.

### Retinal cells survival reduction after 24 h from mouse eyes removal

**Fig. 2:**
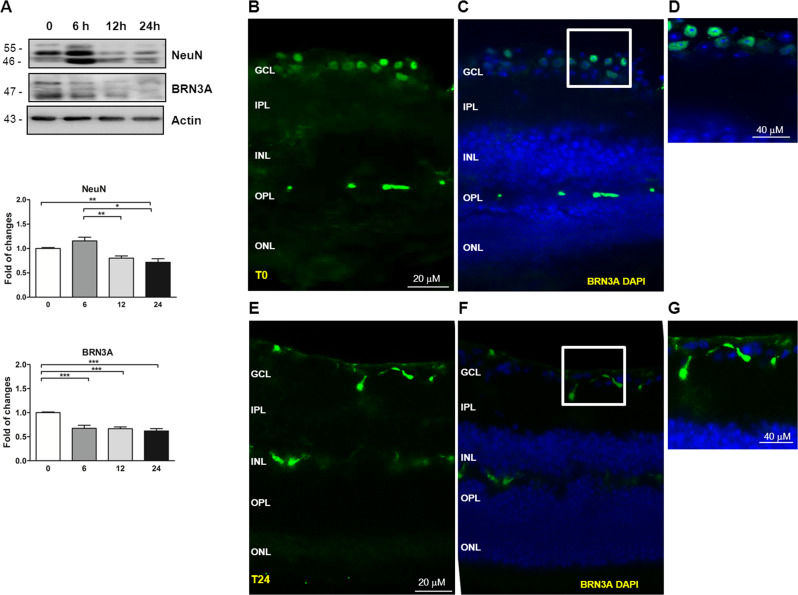
Reduction of retinal ganglion cells and retinal cells vitality after 24 h from C57BL/6J mice eye removal. **A** Representative western blot and relative quantification of retinas lysates showed a significant reduction of BRN3A [a specific marker for RGC nuclei] and NeuN level in retina lysates collected at time 24 in comparison to time 0. Data were expressed as mean ± SEM. One-way ANOVA, Tukey’s post hoc test. *P < 0.05, **P < 0.01, and ***P < 0.001 [*n* = 5]. **B**–**G** Immunofluorescence of retinal sections performed with BRN3A showed a reduction of BRN3A-positive cells (labeled in green) in retinas collected at time 24 (**E**–**G**) vs time 0 (**B**–**D**). Nuclei were stained with the nuclear marker DAPI (blue). GCL ganglion cell layer, IPL inner plexiform layer, INL inner nuclear layer, OPL outer plexiform layer, ONL outer nuclear layer. Scale bar: 20 μm. **D**, **G** Merged images at time 0 (**D**) and time 24 (**G**). **D**, **G** High magnification of merged images. Scale bar: 40 μm.

Retinal cells vitality within 24 after ONC was evaluated by the expression level of the neuronal marker NeuN that was significantly reduced (*t*24: 0.72 ± 0.07, *P* < 0.01 vs *t*0; Fig. 2A), while it was slightly increased at time 6 but not significant.

Moreover, the expression level of BRN3A, a specific marker for RGC nuclei, decreased in the retinas (*t*6: 0.67 ± 0.06, *P* < 0.001 vs *t*0; *t*12: 0.67 ± 0.06, *P* < 0.001 vs *t*0; *t*24: 0.62 ± 0.05, *P* < 0.001 vs *t*0; Fig. 2A). In parallel, the qualitative immunofluorescence images of retinal slices showed a reduction of BRN3a positive after 24 h (in green, Fig. 2E–G) (Fig. 2 B–D).

### MAPK pathways time-dependent changes and Tau expression level

The MAPK (mitogen-activated protein kinase) signaling pathways, c-Jun, N-terminal kinases (JNKs), and extracellular signal-regulated kinases (ERKs), play a critical role in the control of autophagy and apoptosis responses [29]. In line with data reported in literature [30–32], our results showed a clear activation of the apoptotic pathway in the retina.

**Fig. 3:**
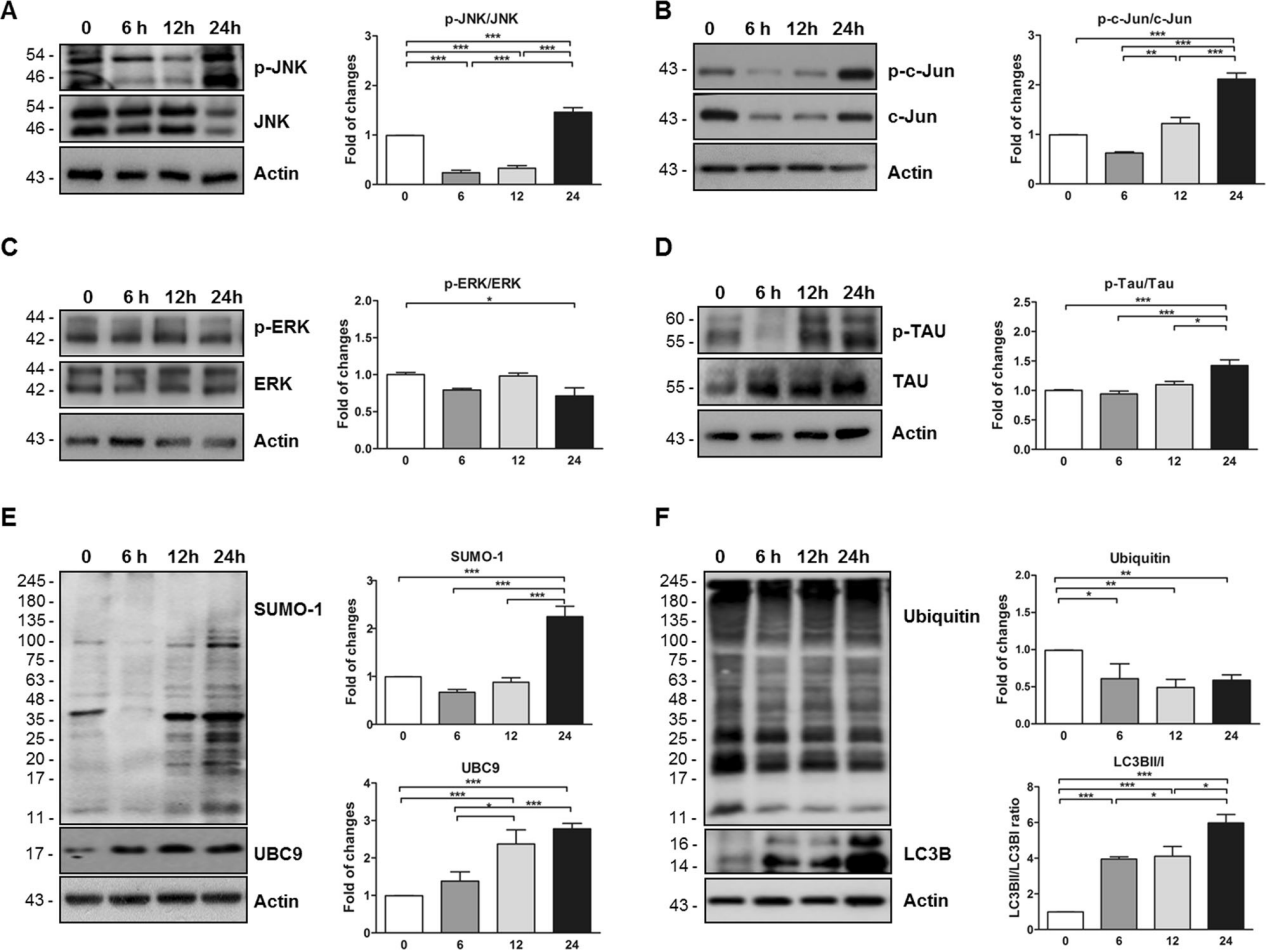
MAPK pathways activation in the retina after 24 h from C57BL/6J mice eye removal. **A**–**D** Representative western blots and relative quantifications showed the significant higher p-JNK/JNK (**A**), p-c-Jun/c-Jun (**B**), and p-Tau/Tau (**D**) ratios in retina homogenates collected at time 24 in comparison to time 0, while the p-ERK/ERK (**C**) ratio was lower. **E** Representative western blots and relative quantifications showed the significant higher SUMO-1 and UBC9 level in retina homogenates collected at time 24 in comparison to time 0. **F** Representative western blots and relative quantifications showed the significant time-dependent decrease of the ubiquitin level and increase of the LC3B level expressed as the LC3BII/I ratio in retina homogenates collected at time 24 in comparison to time 0. *P < 0.05, **P < 0.01, ***P < 0.001, and ****P < 0.0001 [*n* = 5]. Data were expressed as mean ± SEM. One-way ANOVA, Tukey’s post hoc test.

JNK was strongly activated (as p-JNK/JNK) at time 24 (1.46 ± 0.08, *P* < 0.001 vs *t*0; Fig. 3A) but significantly reduced at time 6 (0.24 ± 0.09, *P* < 0.001 vs *t*0; Fig. 3A) and 12 (0.33 ± 0.09, *P* < 0.001 vs *t*0; Fig. 3A). Consequently, the phosphorylation of c-Jun, a transcription factor activated downstream by JNK, increased at time 24 (2.11 ± 0.12; *P* < 0.001 vs *t*0; Fig. 3B). Instead, ERK (as p-ERK/ERK) was slightly reduced at time 24 (0.71 ± 0.09, *P* < 0.05 vs *t*0; Fig. 3C). Tau accumulation in the visual system is an early phenomena of the retinal neuronal damage [18, 33] and interestingly it has a strong crosstalk with JNK in both physiological and pathological conditions [21]. Thus, Tau phosphorylation (as p-Tau/Tau) was analyzed and significantly augmented in retinas extracted at time 24 (1.42 ± 0.07, *P* < 0.001 vs *t*0; Fig. 3D).

### SUMO-1ylation and degradation signaling pathways are altered in the ex vivo model

SUMOylation is known to play an important role in retinal function [22, 34] and in oxidative stress [21]. Interestingly, SUMO-1ylation was highly increased in retinas at time 24 (2.25 ± 0.16, *P* < 0.0001 vs *t*0; Fig. 3E) and the expression level of UBC9, the fundamental SUMOylation enzyme [35], was augmented in a time-dependent manner. Specifically, UBC9 level was increased at time point 12 and 24 (*t*12: 2.37 ± 0.22 and *t*24: 2.77 ± 0.19, *P* < 0.0001 vs *t*0; Fig. 3E).

The failure of the degradation pathway is reported to be involved in several degenerative processes leading to retinal cell dysfunction in eye’s pathologies [36]. Therefore, the ubiquitin and LC3B conversion level was measured in retina homogenates and ubiquitin level was reduced at time 6 (0.61 ± 0.11, *P* < 0.05 vs *t*0; Fig. 3F), at time 12 (0.49 ± 0.11, *P* < 0.01 vs *t*0; Fig. 3F), and time 24 (0.59 ± 0.09, *P* < 0.01 vs *t*0; Fig. 3F), while LC3B conversion (LC3BII/LC3BI ratio) was augmented in each time points: time 6 (3.95 ± 0.44, *P* < 0.0001 vs *t*0; Fig. 3F), time 12 (4.11 ± 0.44, *P* < 0.0001 vs *t*0; Fig. 3F), and time 24 (5.98 ± 0.44, *P* < 0.0001 vs *t*0, Fig. 3F).

### Curcumin treatment prevented RGC loss in our ex vivo model

Curcumin, a natural antioxidant compound largely used to contrast degenerative processes [21, 37], was tested at 5 and 10 μM to counteract the cell death of retinal layers in our ex vivo model at 24 h. Firstly, we verified if curcumin vehicle (DMSO) had toxic effects and likely the levels of cleaved-Caspase-3, BAX, BCL2, and BCL-XL were not altered at time 24 (see Suppl. Fig. 1A).

**Fig. 4:**
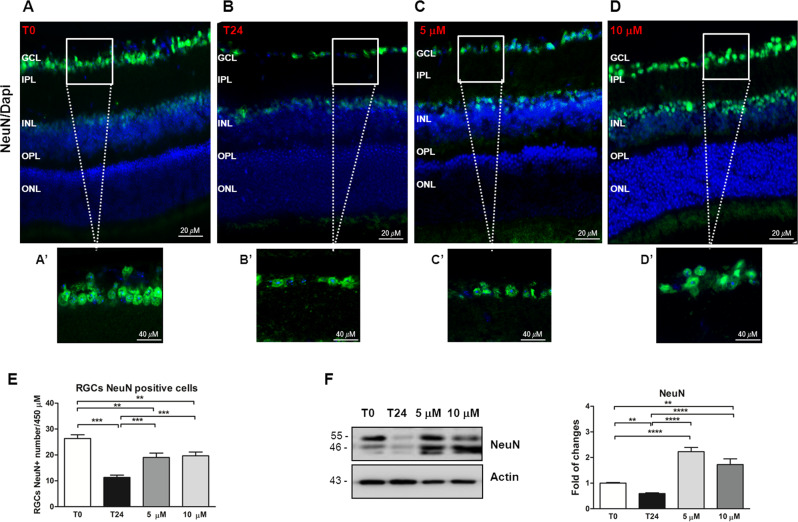
Curcumin treatment reduces the retinal degeneration preventing the RGC loss in our ex vivo model of retinal degeneration. **A**–**D** Immunofluorescence analysis of retinal sections collected immediately after the sacrifice (*T*0), after 24 h after the sacrifice (*T*24), 24 h pre-treated with 5 μM (5 μM) and 10 μM (10 μM) of curcumin. IF images indicated a decrease of RGC NeuN-positive cells (green dots) in samples collected after 24 h from eye removal (**B**–**B**′) compared to time 0 (**A**–**A**′). The 5 μM (**C**–**C**′) as well as 10 μM (**D**–**D**′) of curcumin treatment was able to prevent the RGC loss detected at time 24. Nuclei were stained with the nuclear marker DAPI (blue). Scale Bar: 20 μm. **A**′, **B**′, **C**′, **D**′ High magnification of merged images. Scale bar: 40 μm. GCL ganglion cell layer, IPL inner plexiform layer, INL inner nuclear layer, OPL outer plexiform layer, ONL outer nuclear layer. **E** Quantification of the RGCs NeuN-positive (NeuN+) number showed a reduction of RGC NeuN+ in retinas collected at time 24 compared to time 0. Both 5 and 10 μM curcumin administration was able to reduce RGC loss, increasing significantly RGC NeuN+ cells number. **F** Representative western blot and relative densitometric quantification showed a reduction of NeuN level in retina homogenates collected at time 24 and a significant increased NeuN level induced by the 5 and 10 μM curcumin administrations. **P < 0.01 and ****P < 0.0001 [*n* = 5]. Data were expressed as mean ± SEM. One-way ANOVA, Tukey’s post hoc test.

The immunofluorescence images showed a reduction of NeuN staining at time 24 (labeled in green, Fig. 4A, B) in which the number of NeuN-positive RGCs (Fig. 4A′–B′) were decreased (25.4 ± 1.5 vs 11.80 ± 1.5, *P* < 0.001; Fig. 4E).

The pre-treatment with curcumin reduced RGC loss at 5 μM (19.2 ± 1.3, *P* < 0.001 vs *t*24; Fig. 4E) and 10 μM (20.4 ± 1.3, *P* < 0.0001; Fig. 4E), shown also in the immunofluorescence images (labeled in green, Fig. 4C–C′, D–D′). Importantly, the NeuN expression level was reduced after 24 h (0.72 ± 0.04, *P* < 0.01 vs *t*0; Fig. 4F) while curcumin pre-treatment prevented neuronal loss at both 5 and 10 μM (5 μM: 2.23 ± 0.18; 10 μM: 1.72 ± 0.18, *P* < 0.0001 vs t24; Fig. 4F).

### Curcumin protected the thickness of some retinal cells layer in our ex vivo model

**Fig. 5:**
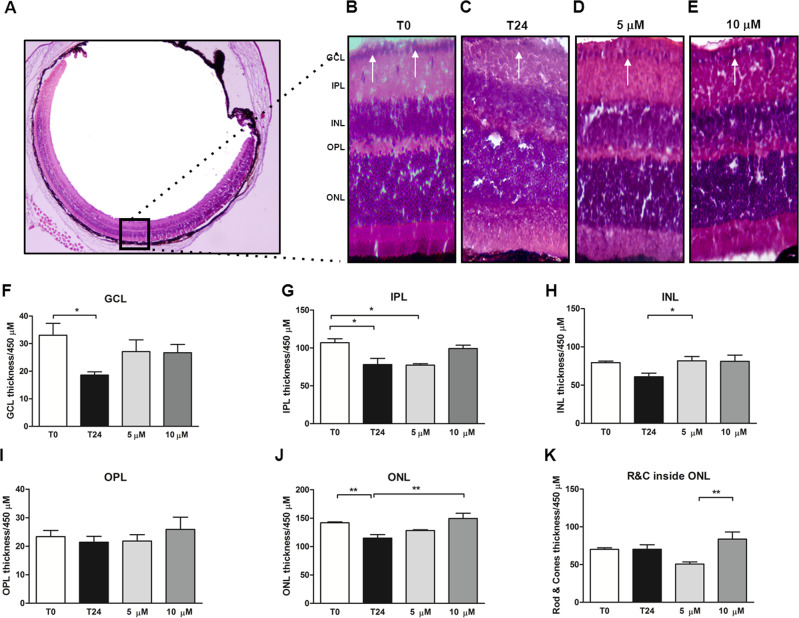
Alterations of retinal layers thickness and influence of curcumin administration in induced retinal degeneration ex vivo model. **A**–**E** Representative image of hematoxylin & eosin (H&E) eye’s sections collected at time 0 (**A**) and magnification of retinal sections examined at time 0 (**B**), time 24 (**C**), 24 h pre-treated with 5 μM (**D**), and 10 μM (**E**) of curcumin depicting retinal morphological changes in H&E staining. GCL ganglion cell layer, IPL inner plexiform layer, INL inner nuclear layer, OPL outer plexiform layer, ONL outer nuclear layer, OS outer segment. Scale bar: 20 μm. **F**–**K** Measurement of retinal layer thickness in H&E eye sections collected at time 0, after 24 h from eye removal, 24 h pre-treated with 5 and 10 μM of curcumin. Graphs indicated at time 24 a reduction of ganglion cell layer (GCL, **F**), inner plexiform layer (IPL, **G**), inner nuclear layer (INL, **H**), outer nuclear layer (ONL, **J**), if compared to time 0. A tendency of increasing thickness was observed in samples pre-treated with curcumin in each layer analyzed, but resulted significant only for INL and ONL analysis (**H**, **J**); graph related to rod and cones inside ONL (R&C, **K**) indicated a significant increase between 5 and 10 μM curcumin-treated samples. The measurement of outer plexiform layer (OPL, **I**) did not show any significant differences among groups examined.*P < 0.05 and **P < 0.01 [*n* = 5]. Data were expressed as mean ± SEM. One-way ANOVA, Tukey’s post hoc test.

The neuroprotective curcumin effect was analyzed by measuring the thickness of the retinas layers (Fig. 5A–E). Interestingly, the reduction of GCL thickness confirmed RGC loss at time 24 (18.62 ± 4.70; *P* < 0.05 vs *t*0; Fig. 5C, F). The curcumin 5 μM (27.13 ± 5.02, Fig. 5D, F) and 10 μM (26.73 ± 5.02, Fig. 5E, F) pre-treatment induced a slight, but not significant, recovery of RGC loss within the 24 h. The IPL thickness was reduced at 24 h (78.10 ± 7.92; *P* < 0.05 vs *t*0; Fig. 5C, G) and the IPL thinning was not prevented by 5 μM curcumin treatment (77.35 ± 8.40, *P* < 0.05 vs *t*0; Fig. 5D, G), while 10 μM induced a slight protection (99.25 ± 8.40, Fig. 5E, G). The INL and outer plexiform (OPL) layer thicknesses were not affected within the 24 h. Interestingly, the outer nuclear layer (ONL) thickness was slightly reduced in 24 h (114.9 ± 6.50, *P* < 0.01 vs *t*0; Fig. 5C, J), prevented with 10 μM curcuma treatment (149.6 ± 6.50, *P* < 0.01 vs *t*24; Fig. 5E, J).

### Curcumin prevented apoptotic pathways and MAPKs activation in the ex vivo model

**Fig. 6:**
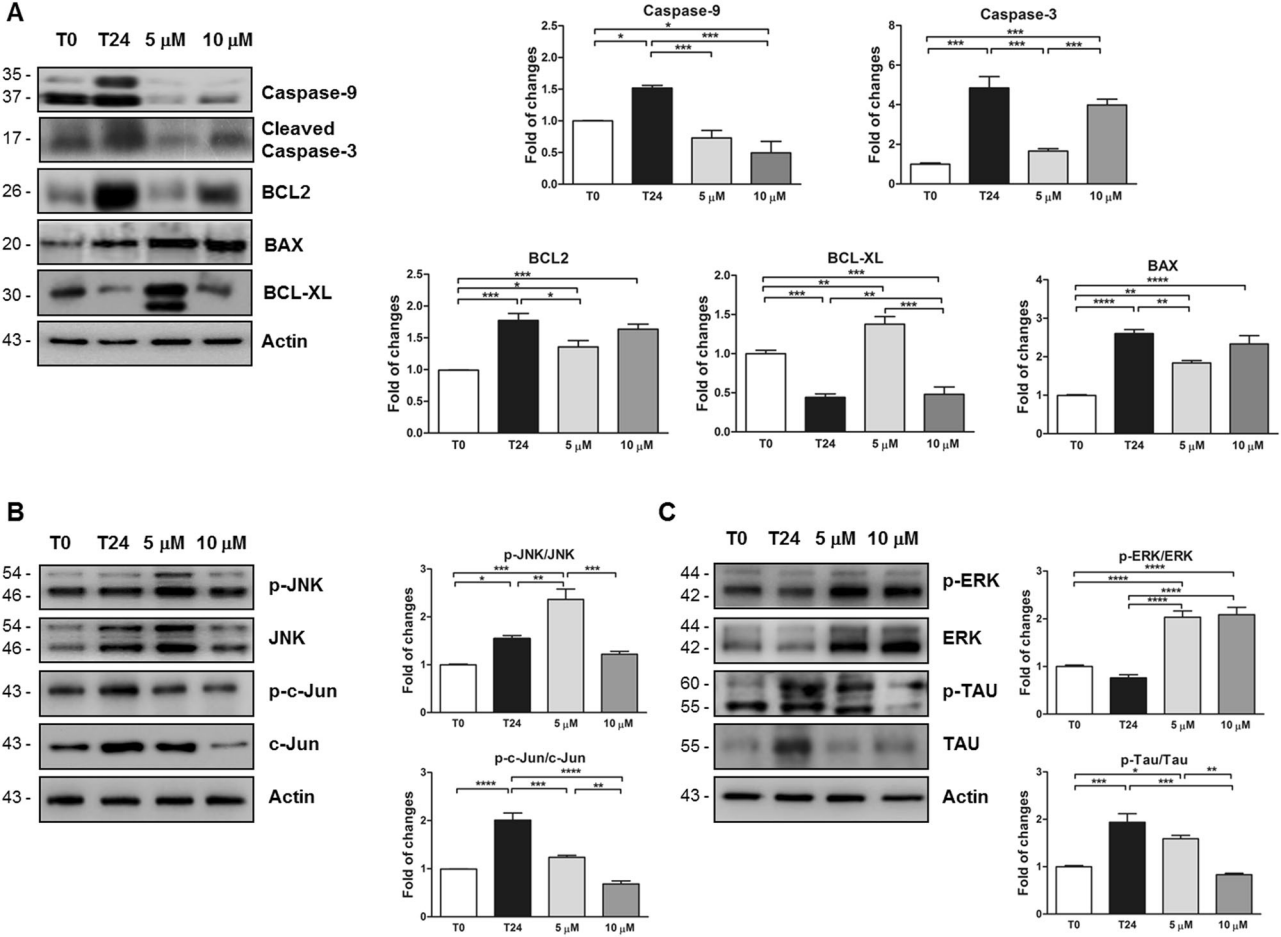
Curcumin treatment prevents apoptotic and MAPK pathways activation in ex vivo model of retinal degeneration. **A** Representative western blots and relative quantifications showed the reduction of Caspase-9, cleaved-Caspases-3, BCL2, and BAX levels in retina homogenates caused by the 24 h pre-treatment of curcumin (5 and 10 μM) if compared to induced retinal degeneration at time 24. The BCL-XL level was significantly increased by 5 μM of curcumin administration. *P < 0.05, **P < 0.01, ***P < 0.001, and ****P < 0.0001 [*n* = 5]. Data were expressed as mean ± SEM. One-way ANOVA, Tukey’s post hoc test. **B**, **C** Representative western blots and relative quantifications of JNK, c-Jun, ERK, and Tau. **B** Graphs indicated a significant augmented p-JNK/JNK and p-c-Jun/C-Jun ratios at time 24, reduced by curcumin administration; 5 μM of curcumin significantly increase p-JNK/JNK ratio if compared to other experimental conditions. **C** A significant decrease of p-ERK/ERK ratio at time 24 was caused by 5 and 10 μM of curcumin administration. The augmented p-Tau/Tau ratio observed at time 24 was reduced by curcumin treatment. *P < 0.05, **P < 0.01, ***P < 0.001, and ****P < 0.0001 [*n* = 5]. Data were expressed as mean ± SEM. One-way ANOVA, Tukey’s post hoc test.

Interestingly, curcumin pre-treatment prevented the activation of the apoptotic pathway in retinal lysates, specifically the expression level of Caspase-9 (0.73 ± 0.15, *P* < 0.001 vs *t*24; Fig. 6A) and Caspase-3 (0.165 ± 0.45; *P* < 0.0001 vs *t*24, Fig. 6A) were both reduced at 5 μM of curcumin, while at 10 μM the Caspase-9 level was decreased (0.49 ± 0.15, *P* < 0.0001 vs *t*24; Fig. 6A) but not the level of cleaved-Caspase-3. This effect is in line with previous studies indicating that 5 μM of curcumin was the most effective dose against degenerative processes [21, 37, 38]. In fact, 10 μM of curcumin was unable to contrast the BCL2 and BAX levels augmentation at time 24 (BCL2: 1.77 ± 0.12; *P* < 0.001 vs *t*0 and BAX: 2.60 ± 0.18, *P* < 0.0001 vs *t*0; Fig. 6A). However, 5 μM of curcumin reduced BCL2 and BAX levels (BCL2: 1.36 ± 0.12, *P* < 0.05 vs *t*24; BAX: 1.83 ± 0.18, *P* < 0.01 vs *t*24; Fig. 6A). In line, the BCL-XL level was decreased at time 24 (0.44 ± 0.09, *P* < 0.0001 vs *t*0; Fig. 6A) and the pre-treatment with 5 μM of curcumin contrasted this effect (1.37 ± 0.09, *P* < 0.001 vs *t*24; Fig. 6A), while 10 μM of curcumin was again ineffective.

Then, JNK phosphorylation was augmented at time 24 (1.54 ± 0.17, *P* < 0.05; Fig. 6B), but surprisingly the pre-treatment with 5 μM of curcumin caused an even more significant increase of p-JNK/JNK ratio (2.36 ± 0.17, *P* < 0.01 vs *t*24; Fig. 6B), while 10 μM of curcumin showed a slight, but not significant, reduction of JNK activation (1.22 ± 0.17, Fig. 6B).

C-Jun activity was strongly increased at time 24 (2.11 ± 0.12, *P* < 0.001 vs *t*0; Fig. 6B) and it was reduced by both doses of curcumin (5 μM: 1.24 ± 0.11, *P* < 0.001; 10 μM: 0.68 ± 0.11, *P* < 0.0001 vs *t*24; Fig. 6B). As expected, ERK activation had an opposite trend respect to JNK and c-Jun showing a decrement of p-ERK/ERK ratio at time 24 that was reverted by both 5 μM (2.03 ± 0.14, *P* < 0.0001 vs *t*24; Fig. 6B) and 10 μM (2.10 ± 0.15, *P* < 0.0001 vs *t*24; Fig. 6B) of curcumin.

Finally, we found that the augmented p-Tau/Tau ratio level caused by the 24 h after eyeballs enucleation (1.94 ± 0.16, *P* < 0.01 vs *t*0; Fig. 6B) was significantly prevented by the pre-treatment of curcumin with by 10 μM of curcumin (0.83 ± 0.16, *P* < 0.0001 vs *t*24; Fig. 6B).

### Curcumin influenced SUMO-1 activity and altered the ubiquitination pathway in our ex vivo model

**Fig. 7:**
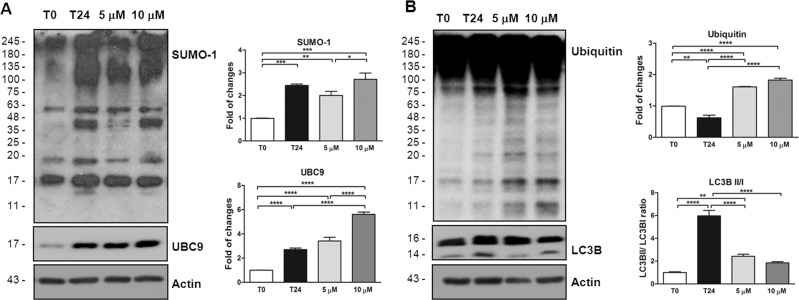
Curcumin influences SUMO-1 and degradation pathway activity in ex vivo model of retinal degeneration. **A**–**D** Representative western blots and relative quantifications of SUMO-1, UBC9, Ubiquitin, and LC3B. **A** Graphs showed an increase of SUMO-1ylated proteins and UBC9 level at time 24 in retinas homogenates if compared to time 0. The treatment with 10 μM of curcumin induced a significant augmentation of SUMO-1 as well as UBC9 levels, while 5 μM caused a slight reduction of SUMO-1 activity. **B** Histograms showed at time 24 a significant increase of Ubiquitin level and a reduction of LC3BII/I ratio induced by 5 and 10 μM curcumin administration. *P < 0.05, **P < 0.01, ***P < 0.001, and ****P < 0.0001 [*n* = 5]. Data were expressed as mean ± SEM. One-way ANOVA, Tukey’s post hoc test.

Then, we investigated the curcumin effect on the SUMOylation and ubiquitination molecular pathways in our ex vivo model finding that protein SUMO-1ylation was increased in the retina lysates at 24 h (2.44 ± 0.20, *P* < 0.001 vs *t*0; Fig. 7A), but the pre-treatment of both doses of curcumin was not able to significantly modify the SUMO-1ylation level.

A similar trend was also observed for UBC9 that was significantly increased at time 24 (2.77 ± 0.19, *P* < 0.0001; Fig. 7A), but curcumin was inefficient to counteract this modification, and actually 10 μM of curcumin increased the level of UBC9 even more than 24 h (10 μM: 5.61 ± 0.25, *P* < 0.0001; Fig. 7A). The expression level of ubiquitin was significantly decreased at time 24 (0.62 ± 0.08, *P* < 0.01 vs *t*0; Fig. 7B) while it was significantly increased by 5 μM (1.60 ± 0.08, *P* < 0.0001; Fig. 7B) and 10 μM of curcumin (1.82 ± 0.08, *P* < 0.0001; Fig. 7B). We tested the effect of curcumin on autophagy system and we found that LC3BII/I ratio was strongly augmented at time 24 (5.98 ± 0.34, *P* < 0.0001; Fig. 7B) and this effect was prevented by both 5 and 10 μM of curcumin administration (5 μM: 2.42 ± 0.34, *P* < 0.0001; 10 μM: 1.86 ± 0.36, *P* < 0.0001 vs t24; Fig. 7B).

## Discussion

In this paper we described an interesting and fast method to study the RGC loss occurring when eyeballs are kept in PBS at 4 °C for 24 h after being enucleated from mice, procedure that includes the ONC. We demonstrated that in 24 h several biochemical parameters related to cells neurodegeneration were altered and curcumin, a turmeric bioactive compound, was efficient to partially contrast the retinal cell loss. Indeed, after 24 h from ONC the optic nerve lysate showed an increase of Caspase-3 and a drop of NeuN levels while retinal lysates showed an increase level of Caspase-9. This let us to suppose that ONC and eyeballs enucleation induced the onset of the apoptotic process which especially leads to RGC loss [39]. Within only 24 h from the ONC and eye isolation a huge increase of BCL2 level, a pro-apoptotic protein, was found in retinal lysate correlated to a significant reduction of BCL-XL, an anti-apoptotic protein, which confirms the activation of apoptotic pathway as already shown for RGCs [40]. Caspase-3 has been indicated as mediator for RGC death following axotomy in vivo [41] while Caspase-7 is involved in an earlier step of the apoptotic signaling cascade in retinal degenerative model [27]. Caspase-7 was found activated in RGCs starting from 12 h post optic nerve crush reaching the plateau after 3 days [27], while Caspase-3 activation was delayed [30]. In our hands, both biochemical and immunofluorescence analysis suggest that our method developed predominantly an important RGC loss, as also shown by the loss of BRN3A immunoreactions, a specific RGC marker.

Then, we found a powerful activation of JNK in retina lysates, which indicates its important role in cell apoptosis as previously described [42, 43].

JNK kinase regulates several transcription factors including c-Jun, but also many mitochondrial proteins like BCL2 and BCL-XL [18, 44]. As expected, being JNK phosphorylation induced by ONC at 24 h also a powerful c-Jun activation was observed, in line with the already known direct interaction cascade between p-JNK and p-c-Jun [45]. In fact, an alteration of BCL2 and BCL-XL levels was detected after 24 h from ONC, probably as a consequence of JNK and apoptosis cascade activation. Interestingly, the expression level of BCL2 and BCL-XL is opposite after 24 h from ONC, in line with the already described interplay between the two proteins acting vicariously for each other in the anti-apoptotic system [46, 47]. The apoptotic induction at 24 h is able to induce the expression of a faint but visible band at around 26 kDa which correspond to the pro-apoptotic isoform BCL-XS [46, 47]. Moreover, a decreased activity of ERK, which counterbalances the JNK activation in apoptotic processes, was observed in the retinas collected after 24 h from ONC, in line with published data [48, 49].

Interestingly, we observed an augmented Tau protein level as reported in animal models affected by Alzheimer’s disease in which an aberrant accumulation of Tau protein in the retina is supposed to cause visual malfunction [18].

In our previous study we proposed a cooperative crosstalk among JNK, Tau, and SUMO-1 in oxidative stress condition [21] and accordingly these proteins have a role in retina function [50]; therefore, we examined the protein SUMO-1ylation in our ex vivo model. We found an increased SUMO-1ylated and UBC9 (SUMO-1 target enzyme) protein level with an opposite trend compared to ubiquitination level after 24 h from ONC which support the retinal suffering. In line with the observation that autophagy is activated in a model of optic nerve crush produced in rats [51] we found an augmented LC3B level in our model. The SUMOylation/ubiquitination process unbalance and the increase of the autophagic system activity suggest a potential engulfment of the degradation pathway which might be the cause for the pathological accumulation of Tau that leading consequently to the Tau hyper-phosphorylation and retinal cell death.

Therefore, we tested curcumin as potential neuroprotective molecule against degenerative processes in this ex vivo model. The therapeutic role of curcumin in retinal diseases has been already hypothesized [25, 26] and we also demonstrated, in a neuroblastoma cell line, that it is able to prevent the SUMO-1–JNK–Tau axis hyperactivation in oxidative stress condition [21]. Indeed, a pre-treatment with 5 and 10 μM of curcumin was able to counteract the activation of the apoptotic pathway, reducing Caspase-9 and Caspase-3 as well as apoptotic (BCL2 and BAX) markers and increased the anti-apoptotic (BCL-XL) level [21]. Specifically, although BCL-XS is activated by pro-apoptotic signals, the treatment with 5 uM of curcumin was able to protect the retinal cells inducing a strong BCL-XL expression. On the other hand, 10 μM of curcumin exerted its retinal neuroprotective effect inducing an augmented expression level of BCL2 protein which vicariate the anti-apoptotic activity of BCL-XL. Probably, curcumin is able to activate two different anti-apoptotic cascades or acts on the same one at different levels depending on the administered concentration.

The protective effects of curcumin against apoptosis and oxidative damage have been already described in different in vitro and in vivo models of glaucoma [52, 53]. In our experiments, curcumin was able to reduce RGC loss when RGCs were counted in NeuN staining and the RGC layer was measured in hematoxylin–eosin staining. As expected, the other retinal layers thickness was comparable with control in both 24 h and curcumin treated indicating that our ex vivo model based on ONC is mainly effecting RGC survival.

In line with our results, pre-clinical studies showed that curcumin administration (2.5 and 10 μM) both in in vitro and in vivo models attenuated RGC and amacrine cell loss [25]. Interestingly, we observed that curcumin seems to re-equilibrate SUMOylation/Ubiquitination balance. Moreover, the strong activation of the autophagic system detected was well prevented by curcumin treatment, suggesting its inhibitory potential on this pathway as already observed [54].

JNK/c-Jun activation and Tau protein level were reduced by curcumin treatment, while ERK activity was significantly increased as already reported in different models of retinopathies [52, 55, 56]. Ten micromolar of curcumin was able to prevent the JNK activation probably for its direct antagonist effect on this kinase [57] especially reducing the JNK3 pro-apoptotic isoform, which can also explain the prevention of the autophagy pathway activation [58]. Unexpectedly, an increased JNK activity was observed after 5 μM of curcumin treatment leading us to hypothesize that curcumin lower concentration is not able to act on JNK pathway, but it may induce the upstream ERK cascade activation. Further studies are to need to better clarify the curcumin effect on the different players of the apoptotic pathway (BCL2, BCL-XL, BAX, ERK, JNK).

Moreover, the activity of curcumin on JNK, reduction of autophagy pathway, and Tau hyperactivation may leads to think could contrast Tau hyper-phosphorylation and eventually Tau accumulation on the retina as described in different pathologies including Alzheimer’s disease [59] or glaucoma [33].

Our findings showed a simple and non-invasive ex vivo model in which the ONC could mimic the main apoptotic events and RGC loss in a short temporal window and that the protective activity of curcumin could be further investigated for eye’s pathologies.

## Materials and methods

### Animals

In this study we used 2-month-old C57BL/6J male mice purchased from Jackson Laboratories, USA. Animals were housed (4 per group) in standard mouse cages, all with (hard wood shavings) as bedding material, ad libitum food and water with a regular 12:12 h light/dark cycle (lights on 07:00 a.m.), at a constant room temperature of 22 ± 2 °C and relative humidity approximately 55 ± 10%.

### Ex vivo treatment

Animals were sacrificed by cervical dislocation and eyes and optic nerves were harvested to investigate the time-dependent retina neurodegeneration and the effect of ex vivo treatments. The enucleated eyeballs were kept at 4 °C in PBS and retinal dissection has been performed immediately after the sacrifice (time 0) or after 6 (time 6), 12 (time 12), and 24 (time 24) hours from the sacrifice. In each experimental condition, retinas were collected [60, 61] and processed for biochemical analysis. At the same time points, total eyes were collected for immunohistochemistry and immunofluorescence analysis (*n* = 5). A different group of animals was dedicated to investigate the curcumin influence on retina neurodegeneration. After the sacrifice, eyeballs with optic nerves were harvested and treated with the vehicle dimethyl sulfoxide (DMSO) (1 μl), curcumin dissolved in DMSO at the concentration of 5 μM (1 μl) or curcumin dissolved in DMSO at the concentration of 10 μM (1 μl). After 24 h of the treatment, retinas and optic nerves were isolated from the eyecup and processed for biochemical analysis (*n* = 5 for each experimental group). Total eyeballs were collected for immunohistochemistry and immunofluorescence analysis (*n* = 5). The definition of curcumin doses was reported by Buccarello et al. [21].

### Preparation of retinal and optic nerve lysates

Retinas and optic nerves were collected and separately lysed in 93 μl of Lysis Buffer solution (LB) composed by 1% Triton X-100 (Sigma-Aldrich, Milan, Italy, nr 9002-93-1), a complete set of protease inhibitors (Complete, Roche Diagnostics, Basel, Switzerland) and phosphatase inhibitors (Sigma, St. Louis, MO), *N*-ethylmaleimide (Sigma-Aldrich, Milan, Italy, nr 128-53-0), a buffer containing the following components (mM): TRIS acetate, 20; sucrose, 0.27; EDTA, 1; EGTA, 1; Na Orthovanadate, 1; NaF, 50; Na Pyrophosphate, 5; Na β- glycerophosphate, 10; and DTT, 1 (Sigma-Aldrich, Milan, Italy). Then samples were kept for 30 min on ice to allow protein extraction. Later a centrifugation step of 10 min at 10,000 r.p.m. was applied to the samples and the supernatant was collected and stored at −20 °C until needed.

### Western blot

Retinal and optic nerve protein concentrations were quantified using the Bradford Assay (Bio-Rad Protein Assay 500-0006, Munchen, Germany); 40 μg of extracted proteins were separated by 10% SDS polyacrylamide gel electrophoresis. PVDF membranes were blocked in Tris-buffered saline (5% non-fat milk powder, 0.1% Tween 20, 1 h, room temperature). Primary antibodies (see Table 1) were diluted in the same buffer (incubation overnight, 4 °C) and described in Table 1. Blots were developed using horseradish peroxidase-conjugated secondary antibodies (anti-Mouse or anti-Rabbit, 1:5000; Santa Cruz Biotechnology, Milan, Italy) and the immunoreactive bands were visualized by exposure to the ECL chemiluminescence system (Cyanagen, westar antares nr XLS142, Bologna, Italy). Actin was used as a loading control for SUMO-1, UBC9, Caspase-3, and Ubiquitin levels. To quantify JNK, c-Jun, ERK activation, and Tau levels we examined the ratio between the phosphorylated and total kinase itself (p-JNK/JNK, p-c-Jun/c-Jun, and p-ERK/ERK ratio) and the phosphorylated/total protein level (p-Tau/Tau ratio). LC3B was quantified analyzing the ratio between LC3BII and LC3BI isoforms. Western blots were quantified by densitometry using ImageJ software and was based on at least three independent experiments.

**Table 1:** Table of antibodies used in the experiments.

Primary antibody	Host animal	Diluition for western blot	Dilution for immunofluorescence	Distributor, Cat, num.
Anti-β-actin	Rabbit	1:5000		Abcam, Cambridge, UK. cat. ab8227
Anti-BAX	Rabbit	1:500		Cell Signaling, Danvers, MA, USA. cat.#5023
Anti-BCL2	Mouse	1:1000		Cell Signaling, Danvers, MA, USA. cat. #15071
Anti-BCL-XL	Rabbit	1:1000		Cell Signaling, Danvers, MA, USA. cat.#2764
Anti-BRN3A	Mouse	1:1000	1:50	Santa Cruz Biotechnology, Milan, Italy (C-20 sc31984)
Anti-Cleaved-Caspase-3	Rabbit	1:1000		Cell Signaling, Danvers, MA, USA. cat. #9654
Anti-Cleaved-Caspase-9	Rabbit	1:1000		Cell Signaling, Danvers, MA, USA. cat. #20750
Anti-p-c-Jun	Rabbit	1:1000		Cell Signaling, Danvers, MA, USA. cat.#9164
Anti-c-Jun	Rabbit	1:1000		Cell Signaling, Danvers, MA, USA. cat. #9165
Anti-p-ERK	Rabbit	1:1000		Cell Signaling, Danvers, MA, USA. cat.#4377
Anti-ERK	Rabbit	1:1000		Cell Signaling, Danvers, MA, USA cat. #4695
Anti-LC3B	Rabbit	1:1000		Cell Signaling, Danvers, MA, USA. cat. #43566
Anti-p-JNK	Rabbit	1:1000		Cell Signaling, Danvers, MA, USA. cat. #9251
Anti-JNK	Rabbit	1:1000		Cell Signaling, Danvers, MA, USA.cat. #9252
Anti-NeuN	Rabbit	1:1000	1:200	Cell Signaling, Danvers, MA, USA. cat. #24307
Anti-SUMO-1	Mouse	1:500		Santa Cruz Biotechnology, Milan, Italy(D-11sc-5308)
Anti-p-Tau	Mouse	1:1000		Thermo Scientific cat.#MN1020
Anti-Tau	Mouse	1:1000		Thermo Scientific cat. #MA5-12808
Anti-UBC9	Rabbit	1:1000		Cell Signaling, Danvers, MA, USA. cat. #4786
Anti-Ubiquitin	Mouse	1:1000		Cell Signaling, Danvers, MA, USA. cat. #3933

### Immunohistochemistry and immunofluorescence analysis

After the sacrifice, the eyes of the animals were fixed in cold methanol:acetic acid:PBS (3:1:4) overnight [62, 63] and were incubated overnight in 30% sucrose before being embedded within optimal cutting temperature compound (OCT, Sigma, St. Louis, MO, USA). Eyes were sectioned at a thickness of 12 μm and eyeballs sections containing the optic disk (four slices per mouse) were used for subsequent analysis. For immunohistochemistry analysis, retinal crio-sections were mounted and stained with hematoxylin–eosin (H&E) (Sigma- Aldrich, see ref. 64), cover‐slipped with Eukitt, and observed under a light-transmission microscope (Nikon) [64].

Cell density in the GCL was determined for each eye by counting the number of cells in the middle part of retina over a distance of 300 μm (200–500 μm from the edge of the optic disc). The thickness of each layers was also evaluated using ImageJ software and compared between eyes harvested at time 0, time 24, and for each curcumin treatment. For immunofluorescence analysis, retinal crio-sections were permeabilized with PBS-Triton X-100 (Fluka) 0.5% for 15 min, washed three times in PBS 1×, and exposed in a blocking solution for 1 h in a humid chamber.

The primary antibody anti-BRN3a and anti-NeuN (see Table 1) was added and incubated overnight at 4 °C. The following day, retinal sections were incubated with Alexa-488 (Green) (1:500; Invitrogen-Thermo Fisher, Milan, Italy) in PBS for 45 min in a humid chamber. Nuclear staining was performed with DAPI (1:500; Invitrogen-Thermo Fisher, Milan, Italy). Coverslips were mounted in Fluorsave mounting medium (Calbiochem, Millipore, Billerica, MA, USA, nr 345789). Positive cells were counted over a distance of 450 μm and four consecutive sections were analyzed [65]. Images were acquired with a Olympus microscope equipped with a Olympus Confocal scan unit (microscope BX61 and Confocal system FV500) managed by AnalySIS Fluoview software with three lasers line, UV diode laser (405 nm), Ar–Kr (488 nm), He–Ne green (546 nm), respectively, used to detect DAPI staining and secondary antibodies. Identical exposure time was used for all the samples. Images measures and analysis were performed by ImageJ software.

### Data analysis

The experiments were repeated at least three times. Statistical analysis was performed using Graph Pad Prism 9 program. WB data and different neuronal counts were analyzed using one-way ANOVA, followed by Tukey’s post hoc test. All data were expressed as mean ± SEM with statistical significance given at *P* < 0.05.

## Supplementary information

S- Fig 1

## Data Availability

The datasets presented in this study can eventually required to the authors. The article has also Supplementary Material.
