# Supplementary material for: Retinal ganglion cell loss in an ex vivo mouse model of optic nerve cut is prevented by curcumin treatment
Source: Cell Death Discov. 2021 Dec 15;7:394. doi: 10.1038/s41420-021-00760-1 (PMC8674341; doi:10.1038/s41420-021-00760-1)
Supplement: Supplementary file 1 — S- Fig 1 [file 41420_2021_760_MOESM1_ESM.doc]

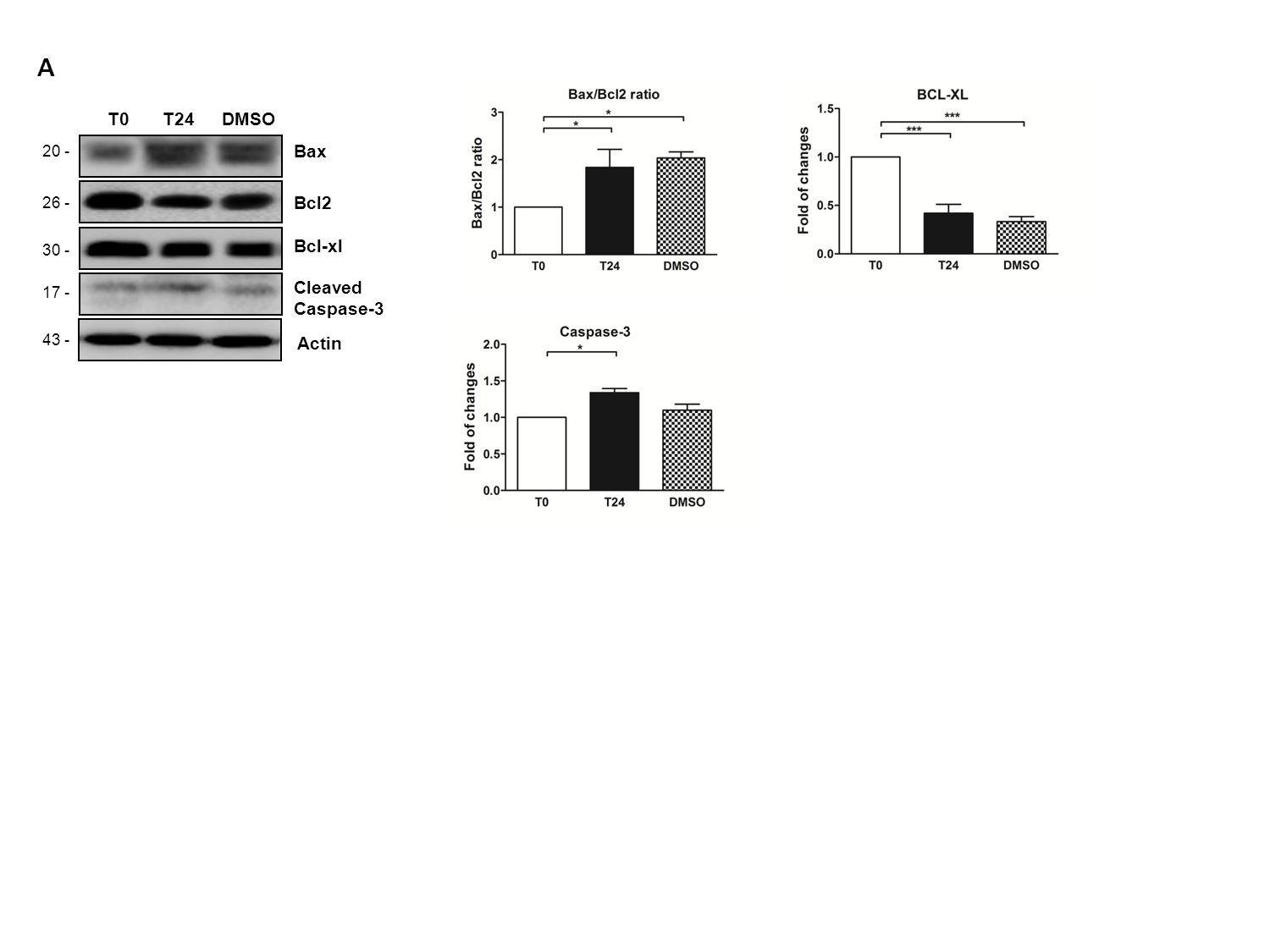


**Supplementary Figure 1. Absence of DMSO effect on retinal degeneration induced by optic nerve cut. A)** Representative western blots and relative quantifications of retinas lysates collected immediately after the sacrifice (time 0), after twenty-four (time 24) hours from the sacrifice and 24 hours pre-treated with DMSO, the vehicle in which curcumin is dissolved. Graphs showed a significant augmented BAX/BCL2 ratio as well as an activation of cleaved-Caspase-3 at time 24 in comparison to time 0. BCL-XL level was decreased in retinas collected at time 24 in comparison to time 0. Any statistical difference was detected comparing retinas collected at time 24 to retinas 24 hours pre-treated with DMSO. Data were expressed as mean ±SEM. One-way ANOVA, Tukey's post-hoc test. *P<0.05 and ***P<0.001 [n=3].
